# Partners or tools? Ethical framings shape welfare research on dogs and horses in animal-assisted services

**DOI:** 10.3389/fvets.2026.1960144

**Published:** 2026-10-19

**Authors:** Friederike Zenker, Karen Thodberg, Peter Sandøe

**Affiliations:** 1 Department of Veterinary and Animal Sciences, University of Copenhagen, Frederiksberg C, Denmark; 2 Department of Animal and Veterinary Sciences, Aarhus University, Tjele, Denmark; 3 Department of Food and Resource Economics, University of Copenhagen, Frederiksberg C, Denmark

**Keywords:** animal ethics, animal welfare, animal-assisted education, animal-assisted services, animal-assisted therapy, one health, positive animal welfare

## Abstract

The welfare of dogs and horses involved in animal-assisted services (AAS) has received increasing scholarly attention, reflecting growing recognition of the ethical complexity of human-animal interactions in these settings. However, ethical framings of animal welfare in research on AAS vary substantially, ranging from an emphasis on avoiding harm to animals while pursuing human benefit to the view that AAS should create mutual benefits for both humans and animals. This critical review examines how ethical framings shape AAS welfare research and aims to make underlying assumptions more transparent to support more consistent research and policy. To this end, we analyzed 116 peer-reviewed papers (2000–2025), focusing on conceptual frameworks (e.g., One Health) and their implications for the interpretation of results and conclusions. Included papers covered direct welfare measures in dogs and/or horses, indirect welfare assessments, and conceptual welfare analyses. We identified three key findings. First, ethical frameworks in AAS welfare research are highly diverse and at times insufficiently specified. Second, studies may combine multiple ethical framings, reflecting a pluralism of ethical perspectives. Conceptual conflation may arise, however, when positive and negative welfare perspectives are combined without acknowledging their fundamentally different understandings of welfare. Third, a recurring pattern across the literature is a relational framing of ethical considerations, highlighting the need for greater attention to different understandings of good or justified human-animal relationships. Overall, greater transparency about ethical framing is needed to improve the interpretation, comparability, and communication of welfare findings.

## 1 Introduction

### 1.1 Ethical framing of animal-assisted services

Animal-assisted services (AAS) are broadly defined as structured practices that involve animals with the aim to support human health and wellbeing (1). Situated within professional practice, these forms of human-animal interaction are comparatively recent in the history of human-animal relations (2). Although horses and dogs—the species most commonly involved in AAS—are recognized as complex sentient beings and are often described as partners (3, 4), AAS remain oriented toward human outcomes and are shaped by economic and organizational constraints (5, 6). Consequently, the animals can be embedded in regimes of training, management, scheduling, and expectations of prosocial behaviors that may position them in an instrumental role with limited control over their participation (4, 7).

Against this background, a growing body of research examines how animals experience AAS (8, 9). Yet evaluating and interpreting findings from animal welfare assessments is not solely a matter of sound science; it also draws on normative claims, including judgments about the ethical legitimacy of AAS [(10), p. 7]. On the one hand, the valued status of the animals involved may situate them within a companion animal framework. Companion animal ethics is characterized by the general assumption that human-animal relationships are potentially mutual, where social animals enrich human lives while also benefiting from care and contact (11). Importantly, this framework also critically assesses misalignments between human perceptions and animals' actual lived experiences. From this perspective, AAS are seen as potentially promoting a good life for the animals. On the other hand, the incorporation of dogs and horses in educational and health-related settings has also been subject to a strand of ethical scrutiny highlighting potential risks and harms to the animals. Involvement in AAS is associated with restrictions and potentially stressful interactions for the animals, including frequent exposure to novel environments and people (12). From this perspective, the animals are in human service, potentially at their own expense (13). This perspective is similar to how the use of animals for human health benefits—such as in biomedical laboratory research—is often viewed. Here, the focus is not on mutual benefit but rather on the avoidance of pain, stress, and other forms of harm to the animals involved, with the goal of reducing and replacing their use as far as possible (14). Taking this approach, AAS at best represents an activity where the animals do not suffer, but from which they do not meaningfully benefit. These two framings are not exhaustive but serve to illustrate how ethical perspectives on AAS can differ substantially and how these differences may shape understandings of ethically justified human–animal interactions. Other plausible framings, such as accounts of human–animal working relations, may relate to these perspectives in complex ways. Such approaches may, for instance, regard mutual benefit as contributing to the justification of animal work (15).

### 1.2 Animal welfare research on AAS

While calls for science-based assessments of animal experiences in AAS abound, welfare research in this field remains relatively recent compared to studies on human outcomes (16). Current welfare assessments rely on both direct measures, such as behavioral and physiological indicators (17, 18), and indirect measures (19, 20), such as handler-reported identification of animal stress, as well as resource- and management-based welfare indicators. Reviews and conceptual papers (21, 22) complement the AAS literature focusing on animal outcomes.

This growing body of welfare studies reports heterogeneous findings, variously described in the literature as limited (23), difficult to compare (9), or even contradictory (24). While some studies raise welfare concerns for horses and dogs across fields of engagement (23, 25–29), others report no acute or chronic negative effects on the animals (23, 30–32). Mendonça et al. (33) find that horses do not experience AAS as either positive or negative.

Common welfare risks mentioned in the literature include animal stress arising from exposure to challenging environments, unfamiliar objects and people, physical restrictions, and work-related demands (12). Frequently, the characteristics of participants with diagnosed health conditions are mentioned as potentially affecting the animals' emotional state in a negative way (34, 35). Studies directed at potential animal welfare benefits expect positive effects from human-animal interactions, such as a relaxed state following activities (36) or elevated oxytocin levels (37), and the provision of cognitively and emotionally rewarding activities, including play (38, 39).

Overall, the heterogeneity of equine and canine welfare evaluations in AAS is, in part, a reflection of the heterogeneous nature of the practices themselves (21). At the same time, this variability underscores the need for more rigorous and systematic analyses to better assess AAS practices from an animal ethics perspective, that is, with explicit consideration of how animals' interests and wellbeing are understood, conceptualized, and negotiated (40).

### 1.3 Aim and review design

Against the presented background, this topic calls for a critical review, as these are particularly suited to bodies of literature that lack conceptual coherence and can benefit from being reconsidered through a new analytical lens (41, 42). The aim of the present review is therefore to trace how the wellbeing of equine and canine actors in AAS is framed and represented in the literature.

Previous critical reviews on the state of equine and canine welfare in the field of AAS have primarily focused on methodological quality, sample size, and outcome validity (33, 43, 44). While of the utmost importance, these focus areas alone cannot fully explain the observed heterogeneity. To date, there is no systematic mapping of ethical framing in AAS welfare research involving dogs and horses. Our review addresses this gap and proposes ethical framing as an additional lens of analysis, arguing that ethical framing shapes research questions, methods, and interpretations in AAS welfare studies, thereby affecting the transparency, reflexivity, and comparability of the research field.

According to van der Staay et al. [(40) p. 1], ethical frames “explicate the underlying moral position” of animal welfare concepts (such as One Health) and welfare assessment frameworks (such as the Five Domains). In this review, ethical frames are understood broadly as conceptual lenses for examining the ethical dimensions of human-animal interactions and practices. Analyses of these frames will include established ethical theories and focus on context-specific ethically laden articulations (40, 45, 46).

## 2 Methodology

### 2.1 Scope of literature & search strategy

The central inclusion criteria for the search were that the papers should explicitly address animal welfare and/or ethics in relation to animal-assisted services or interventions, including the specific practices encompassed by this umbrella term, namely animal-assisted therapy, animal-assisted education, and animal-assisted activities involving dogs and/or horses. Studies were excluded if they were not published in English, primarily focused on human outcomes, focused on dogs placed in clients' homes (such as assistance, guide, or emotional support dogs), or focused on species other than dogs and horses. Search terms were developed based on current terminological conventions in the field (1) and were used in Web of Science and Scopus. Our rationale for using these databases was to cover studies in veterinary, natural and biomedical sciences through Web of Science, alongside also including studies in the social sciences and humanities through Scopus. A limitation is the exclusion of gray literature, which may introduce some bias. Searches were conducted independently by two authors and also covered assistance-dog literature, which was excluded from the present analysis: (“animal-assisted” OR “equine-assisted” OR “service animal” OR “assistance animal” OR “therapy animal” OR “guide dog”) AND “animal welfare” AND (dog OR horse). The papers were classified into three categories using an author-developed scoring system: 3 = direct welfare assessment (animal-based behavioral, biological, and physiological indicators), 2 = indirect welfare assessment (survey studies, resource- or management-based indicators), and 1 = conceptual or ethical discussions, including reviews. The distinction between categories 3 and 2 is based on the common distinction in animal welfare science between animal-based measures and measures of animals' living conditions, such as management or environmental factors. Category 1 was added for reviews and conceptual papers that discussed animal welfare in AAS without directly assessing it.

### 2.2 Analytical approach

We conducted an animal ethics analysis of the included papers, drawing on conceptual analysis to examine how the animals were considered ethically, and how their lived experiences were framed and evaluated in the context of AAS welfare research (46). A key analytical axis concerned how the welfare of the dogs and horses involved in AAS was positioned along a continuum from an animal welfare ideal that focuses on the absence of pain and stress in animals—commonly referred to as negative animal welfare (40)—to positive animal welfare, which “can be defined as the animal flourishing through the experience of predominantly positive mental states and the development of competence and resilience” [(47), p. 1]. Since there is no consensus on definitions within the field (43), we defined the underlying principles guiding the analytical approach of this paper as follows: negative welfare primarily follows the principle of non-maleficence (also known as the “do-no-harm principle”) (14), whereas positive welfare adds the principle of beneficence to all involved. These map neatly on to the two ethical frameworks mentioned above: a mutuality-oriented companion animal framework and a model focused on harm minimization or avoidance.

In addition, we examined how ethical framing was applied across studies, with particular attention paid to the degree of explicitness, the language, terminology, and concepts employed, and the presence of inconsistencies in its application. We further identified overarching themes of ethical framing, including whether the rationale for studying animal welfare was articulated primarily in terms of the animals' interests and wellbeing or in terms of human interests, such as client safety and the effectiveness of practices, as a mixed rationale, or whether no explicit rationale was stated or the rationale remained indeterminate. Studies were counted within these categories and reported descriptively; no statistical tests were conducted.

As this review provides an exploratory mapping of ethical framing in AAS welfare research, the analysis was primarily interpretive rather than quantitative, following the broader distinction between aggregative and interpretive approaches to literature synthesis (48). Ethical framing was examined across several dimensions, including explicit reference to ethical concepts or frameworks, underlying forms of reasoning, and the ways in which ethical assumptions were expressed through terminology and conceptual language. These dimensions were not treated as mutually exclusive categories, as several could be present within the same paper. The analysis therefore focused on identifying recurring patterns, combinations, and tensions in ethical framing across the literature rather than on assigning each study to a single category or determining the prevalence of individual framings.

This study did not assess the methodological validity of welfare research designs. Specifically, we did not examine whether the ethical framing articulated was supported by a scientifically robust study design. We therefore did not assess methodological challenges, such as operationalizing the assessment of positive emotional states.

### 2.3 Reflexivity statement

The authors come to this study from different disciplinary backgrounds (animal ethics/philosophy, animal welfare research/philosophy, and applied ethology) and bring differing approaches to normative questions of human–animal relations, ranging from pragmatic and welfare-oriented to rights-based perspectives. Despite these differences, we are a team that highly values mutual exchange and each other's perspectives and find it productive to discuss our sometimes differing views on animal welfare and ethics. One of us has prior personal experience with AAS through volunteering, while another has done research on nursing home residents' response to dog visits; however, this was not considered to have substantively informed the analysis presented here.

## 3 Results

After screening the titles and abstracts of the initial search results via Scopus and Web of Science, we assessed 176 full-text articles for inclusion in the study. A total of 116 peer-reviewed studies published between 2000 and 2025 met the inclusion criteria and were considered in the review. Among these studies, the focus was approximately evenly distributed between horse welfare/ethics (52 papers) and dog welfare/ethics (56 papers). Eight papers considered both canine and equine issues. Concerning the type of practices, 55 studies framed their research using a broad umbrella term such as animal-assisted interventions or animal-assisted services, 48 focused on therapeutic contexts (although the specific approaches and aims varied significantly), seven studies dealt with educational settings, and six studies with animal-assisted activities. The majority of studies (60 papers) measured animal welfare directly. A total of 25 papers used indirect measurements, and 31 papers were classified as reviews and conceptually driven papers. Overall, we encountered a significant heterogeneity concerning contexts and practices, for example mounted and unmounted practices in the field of equine-assisted services, and dogs used for individual versus group therapy sessions, such as in schools or programs in prisons.

### 3.1 Ethical frameworks identified in the literature

#### 3.1.1 Established reference frameworks

The reviewed literature engages with ethical frameworks spanning the continuum from negative to positive animal welfare. Within our sample, a characteristic expression of negative welfare framing was a focus on assessments of acute or chronic stress in the animals involved in AAS (17, 49–56). Positive animal welfare has emerged as a distinct ethical perspective in AAS research, particularly in recent studies. (36, 39, 57–59). Miller et al. (39), for example, argue for greater attention to assessing positive therapy dog welfare and situate this development within broader conceptual developments in animal welfare science, embracing several elements discussed in the literature, such as quality of life and positive emotions. Overall, negative and positive welfare perspectives are not mutually exclusive. Rather, one approach evident across studies is to explicitly investigate both negative and positive states of animals participating in AAS (37, 57, 60, 61).

One welfare assessment framework cited and applied across the reviewed literature is the Five Domains model (9, 34, 51, 57, 62–64). Contemporary versions of this framework recognize the full spectrum of animal welfare states from negative to positive.

Hall et al. (65), McKinney et al. (31), and Sarrafchi et al. (58) refer to Quality of Life as an ethical reference point. Originally developed within human health care and later adapted in veterinary medicine to assess disease progression in dogs, the concept is now used more broadly in veterinary science to encompass both positive and negative welfare aspects (66), for example by referring to physical condition, emotional states, and opportunities for species-specific behavior (67).

Another framing evident in recent AAS research is the One Health approach (16, 18, 68–72). One Health emphasizes the close interconnections among human, animal, and planetary health (73). Within the reviewed literature, One Health is invoked with varying degrees of specificity, and in some cases remains a relatively broad reference point. A more specific formulation, however, presents well-structured AAS as potentially promoting human and animal health and flourishing simultaneously, rather than positioning their interests as necessarily involving trade-offs (16, 74, 75). Mignot et al. (35) further emphasize this perspective by drawing explicitly on the One Welfare concept derived from One Health and identifying robust benefits for the dog as a minimum requirement for an ethical justification of AAS.

A small number of studies explicitly draw on ethical frameworks originating in studies on the use of laboratory animals. A particularly clear example is provided by Simonato et al. (13), who approach animal welfare in AAS through the 3Rs framework. Within this framework, the aim is to minimize harm (Refinement), reduce animal involvement (Reduction), and consider alternatives to animal involvement (Replacement). The authors discuss the strengths and limitations of each of the 3Rs in the context of AAS [(13), p. 411]. Sullivan et al. (76) and Winkle et al. (77) refer to principles from animal research ethics as one lens among several for addressing animal welfare in AAS. In a less direct sense, Brelsford et al. (78) emphasize the need to better understand the human outcomes of AAS in order to avoid involving animals in interventions unnecessarily.

#### 3.1.2 Adapted and newly articulated ethical frameworks

The reviewed literature also contains isolated examples of newly articulated ethical frameworks. An example is the concept of *symbiosis*, borrowed from biology and employed by Fournier et al. [(79), p. 2] as a cost-benefit model for evaluating human-horse relations in equine-assisted services as transactional relations.

We also found references to conceptual frameworks newly introduced into the AAS literature that remain relatively distinct, e.g. the Capability Approach (80) and Interpretative Phenomenological Analysis (81). Other looser, less technically defined frameworks include reference to the notion of “a life worth living” (62), “a good life” for horses (58), a cost-benefit formula (82), and in contrast a rejection of any trade-offs in which animal wellbeing is compromised for human gain (16, 83, 84). Furthermore, consent (85), animal agency (58), and self-efficacy (71) were brought into the discussion as important concepts with ethical significance. For example, Sarrafchi et al. [(85), p. 2] place questions of consent in the broader context of what constitutes an ethically legitimate relationship, contrasting “consensual interaction” with being “part of an exploitative relationship.” In the study by Naber et al. [(71), p. 2], the use of the concepts self-efficacy and self-expression appear to acknowledge animal agency in a particular way and are described as “fundamental needs for both humans and animals,” and “crucial concepts in reducing stress.” In their study, the authors note that these concepts are typically articulated in human-centered ways and that their application to other animals remains debated.

While strictly speaking not ethical frameworks, legal regulations also serve as a point of departure for addressing animal welfare in parts of the AAS literature. For example, Ameli et al. [(19), p. 2] refer to the German Animal Welfare Act to frame animal welfare in terms of human responsibility for “animals as fellow creatures whose lives and wellbeing must be protected. No one,” according to the authors, “may inflict pain, suffering, or harm on an animal without reasonable cause.” In a similar vein, some studies cite professional guidelines in the field as an ethical foundation for their research, e.g. the IAHAIO White Paper (50, 83).

#### 3.1.3 Ethical assumptions embedded in language and terminology

Across the reviewed studies, we observed that choice of words and terminology reflected normative assumptions about the roles assigned to animals within human–animal interactions (86). A first example concerns therapy-first language, recommended by Wood et al. (87), which foregrounds the licensed therapist instead of the animal, for example in articulations such as “psychotherapy incorporating horses,” used by Friend et al. (88). In contrast, the idea of animals as “co-therapists” (68) conceptually aligns the roles of human and animal more closely. This second category also encompassed a range of paraphrased formulations, including animals “providing a huge service for many people in need” [(28), p. 237], indicating a valuable societal role (89).

Language with strong implications about the social nature of dogs and horses involved in AAS was also evident, for example, in references to dogs as “social butterflies” [(90), p. 9] and “natural traits of love and sociability” [(77), p. 2]. In addition, AAS were referred to as a potentially “meaningful and fulfilling activity” from the animal's point of view [(91), p. 1]. In one example, horses were referred to as “emotional mediators” [(53), p. 11].

At the other end of the spectrum, animals were explicitly referred to as a “therapeutic tool” [(55), p. 18]. Differences were also apparent in the language used to describe animal involvement in AAS. The word “use” was chosen in some studies as a descriptive term (92), while in others the authors distanced themselves from it by placing the term in quotation marks [(77), p. 6]. Elsewhere, animals were described as being “integrated,” “involved,” “engaged,” or “participating” in AAS (4, 19, 71).

### 3.2 How ethical frameworks were applied

#### 3.2.1 Human- and animal-centered rationales

The rationales expressed for studying animal welfare in the context of AAS ranged from those centered on the animals' interests (13 papers; 11.2%) to those focused predominantly on human concerns (8 papers; 6.9%), with 32 papers (27.6%) adopting approaches that combined both perspectives to varying extents. In 63 papers (54.3%), the underlying rationale could not be clearly identified. This does not indicate an absence of an ethical rationale; rather, the rationale was not sufficiently explicit to support a clear classification.

Human-centered rationales concerned, for example, studying animal welfare as a means to improve animal performance (93, 94), therapeutic or educational outcomes (68, 95–97), client safety (98), and overall organizational efficiency (68, 96).

By contrast, animal-centered rationales focused on understanding the animal's affective states for their own sake and safeguarding their health, physical integrity (89), and overall wellbeing (19, 20).

#### 3.2.2 Framework application across individual studies

Overall, the application of ethical framing within individual studies was consistent. Ethical frames introduced in a study typically aligned with the concepts used in the methodology and analysis. For example, studies grounded in a positive welfare framework typically interpreted their results in the discussion and conclusion through an animal-benefit-oriented lens (33, 36, 37) and did so in a consistent way, even when the empirical findings indicated that the intervention in question (e.g., brushing a horse) did not, in fact, provide a direct benefit to the animal (59). Similarly, studies grounded in a negative welfare framework assessed AAS primarily with reference to the absence of stress and other harm, rather than in terms of potential benefits and flourishing (17).

However, we also identified tensions within individual studies' applications of ethical frames. One conceptual issue we observed was the conflation of positive and negative welfare framings. Conceptually, claims of positive animal welfare require robust evidence of short- and/or long-term positive affective states (39) and/or overall flourishing (47), rather than the mere absence of harm or stress, as is sufficient within a negative welfare framing. In some studies, positive welfare—or related notions such as animal wellbeing or their enjoyment of being involved—was introduced as a guiding principle in the introduction, with conclusions subsequently framed in such terms. However, the findings presented in the results and discussion were generally limited to the absence/minimal occurrence of stress indicators or the reporting of stable physiological measures (50, 90, 91). Thus, there was no substantial assessment of robust benefits to the animals. This discord was also reflected in sections where claims of mutual benefit coexisted with reference to welfare assessments that were limited to negative indicators, thereby blurring the conceptual distinction between the absence of harm and the presence of positive welfare [(65), p. 17; (99), p. 69].

### 3.3 Overall patterns in ethical framing

#### 3.3.1 Comparing welfare assessments of dogs and horses

Some studies involving horses compared their welfare during “standard” riding or “leisure riding” and AAS (31, 50, 55, 57). One conceptual paper used a comparative welfare lens, comparing therapy dogs and other companion dogs (100).

When comparing species-specific ethical framing, it became apparent that general welfare concerns regarding equine husbandry, handling, and riding practices were sometimes brought into question. Horse wellbeing in AAS contexts, as some papers argue, cannot be assessed independently of the broader welfare issues associated with keeping and riding horses, such as restrictions on movement, autonomy, and social structures (4, 101). Individual studies raised the concern that some horses in AAS contexts may have developed “learned helplessness,” defined as “a state where they have lost their ability to react to negative stimuli” [(101), p. 2]. This was cited as a factor that may limit the validity of behavioral stress assessments in equine-assisted services. The effect of training was also mentioned with regard to dogs, suggesting that they may maintain composure while displaying more subtle signs of stress (85).

#### 3.3.2 The triad as an overarching analytical lens

One model that appeared across the reviewed studies as a key framework for discussing ethical issues was the triadic handler-animal-client model, which conceptualizes the relationships between client and handler, handler and animal, and animal and client (8).

Within this framework, studies discussed questions of responsibility, particularly who is responsible for recognizing and addressing the animal's needs (62). Ameli et al. [(49), p. 2] provide an example by highlighting the role of the professional practitioner as “the most significant role” within the triad for ensuring animal welfare. Studies also addressed the potential risks of interactions between animals and clients (35), as well as possible positive synergistic effects and cooperation (102). In addition, several studies emphasized the importance of the quality of individual relationships within the triad (4, 18).

## 4 Discussion

A key insight to emerge from this review is that ethical frameworks informing AAS welfare research are highly diverse and articulated with varying degrees of explicitness and at different analytical levels. Furthermore, ethical frameworks can overlap within individual studies. While multiple ethical framings within a single study may reflect a legitimate pluralism of ethical perspectives, their combination may also lead to conceptual conflation. This seems especially relevant when there is a mismatch^1^ between the ethical framing adopted and the aspects actually assessed, for example when only stress is assessed as a welfare indicator in a paper otherwise framed in terms of positive welfare. In addition, despite diverse ethical frameworks a relational perspective is evident across the literature, focusing on relationships, responsibilities, and human-animal interactions. The type and quality of specific human-animal relationships emerge as important considerations for ethically justifying the inclusion of animals in AAS from an animal-centered perspective.

### 4.1 Diversity of frameworks

The diversity of ethical frameworks identified in this review can partly be explained by the heterogeneity of the literature included, which reflects diverse theoretical and practical considerations. As suggested by Fragoso et al. (103), how animal welfare is conceptualized in the literature may reflect not only *prima facie* ethical orientations but also pragmatic or strategic considerations, for example regarding targeted audiences.

Beyond these factors, the diverse frameworks suggest that the literature is shaped by differing ethical assumptions about human-animal relations. As Ghai et al. [(104), p. 672] phrase it, “depending on where one lands on the philosophical spectrum, some may view all [AAS] as exploitative, whereas others may take a more nuanced approach in that [AAS] is viewed as acceptable under certain conditions.” While some studies emphasized the possible alignment of human and animal benefits through AAS, others focused primarily on the absence of stress or other negative states in the animals.

Positions on the acceptability of animal use constitute one central axis along which ethical approaches toward animals differ [(105), p. 5]. Animal welfare science is often characterized as operating from the assumption that animal use for human benefit is acceptable, provided that welfare conditions are met (106). The AAS literature on dog and horse welfare, however, appears to present a somewhat more ambiguous picture (107). One position evident across the literature is that AAS constitutes a form of animal use that may be ethically justified provided that major animal welfare concerns are avoided. For example, Ayala et al. [(50), p. 13] conclude that equine-assisted therapy is justified where it benefits clients without producing stress in horses “different from that produced by any activity or external stimulus.” At the same time, the normative language used in some studies suggests a robust shift away from instrumental concepts (see 3.1.3. in this review). Instead, animals are increasingly described as relational partners (27), and some studies adopt demanding positive-welfare framings that set a high bar for ethically legitimate practice, emphasizing mutual benefit and integrative perspectives such as One Welfare. Lastly, in contrast to perspectives that question or reject animal use altogether, the reviewed literature gives little attention to whether AAS may be problematic in ways that would imply replacing animals, for example with robotic animals, or abandoning such practices altogether.

Making ethical assumptions more transparent may help clarify how AAS welfare studies position themselves along key ethical axes, including the spectrum from negative to positive welfare and the permissibility of animal use. Greater clarity in this regard may also improve comparability across studies and support argumentative coherence, including how welfare indicators such as stress are interpreted. Figures 1, 2 illustrate two selected analytical axes to which we recommend greater attention in AAS welfare research: negative and positive welfare framings and differing rationales for studying welfare. These are not intended to exhaust the relevant ethical dimensions, many of which overlap and do not lend themselves to similarly straightforward visualization.

**Figure 1:**
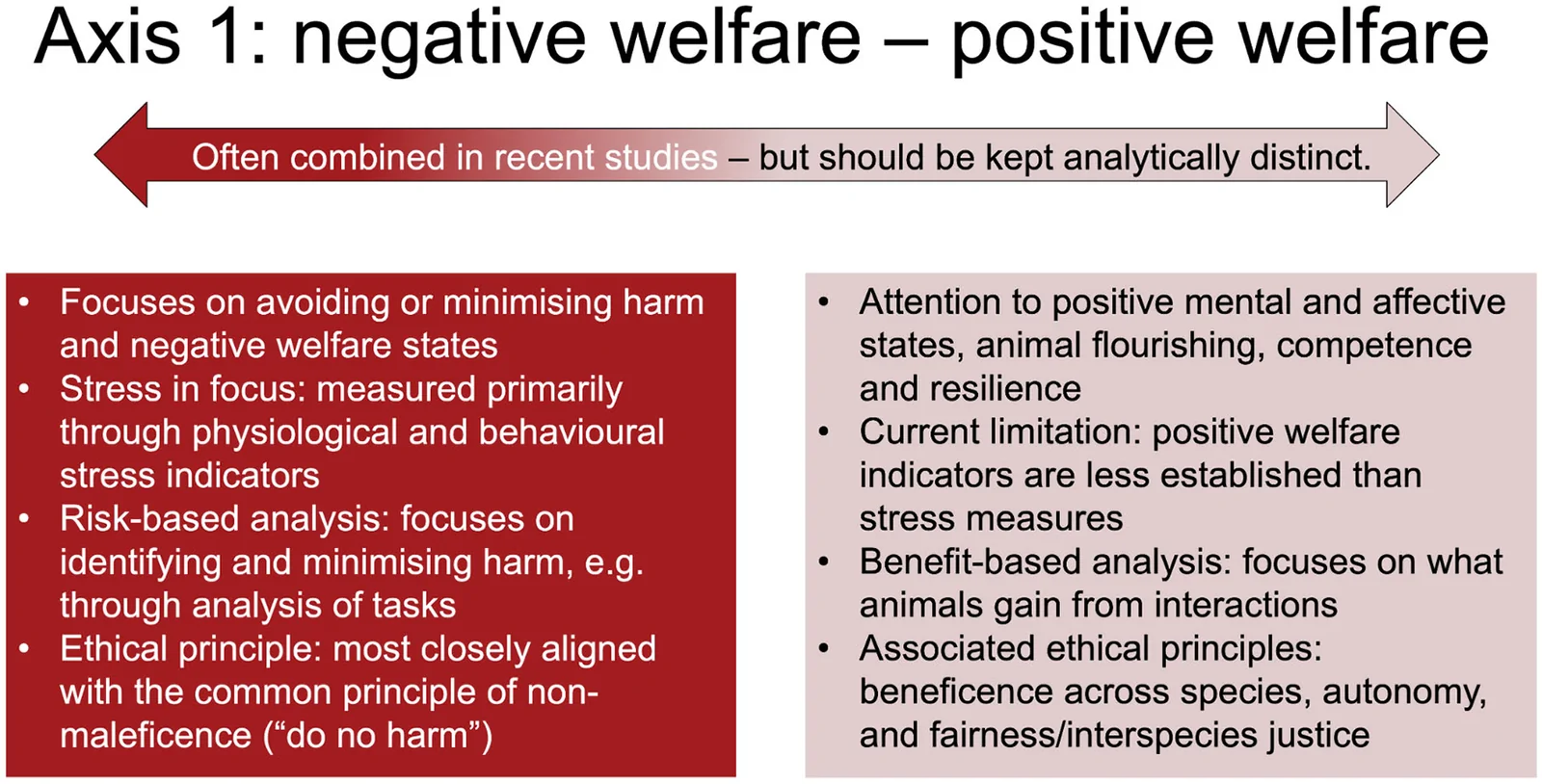
Positive and negative welfare framings as an analytical axis in AAS welfare research. The figure illustrates differences between approaches focused on avoiding or minimizing negative welfare states and those attending to positive welfare states. We suggest that these framings be considered explicitly and kept analytically distinct to avoid conceptual conflation, particularly where both are addressed within the same study.

**Figure 2:**
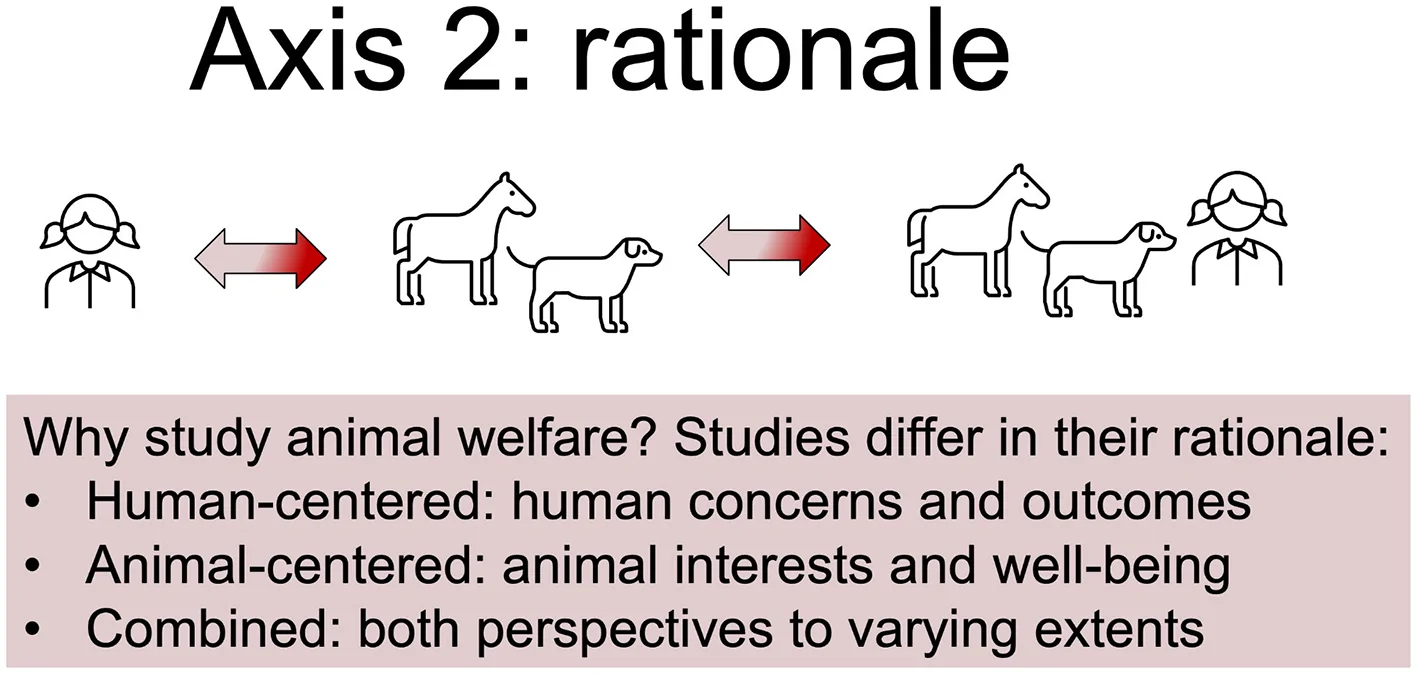
Rationales for studying animal welfare as an analytical axis in AAS welfare research. The figure illustrates different ethical framings of the rationale for studying animal welfare: human-centered, animal-centered, and combined. We suggest that these rationales should be made more explicit as a matter of transparency and reflexivity.

### 4.2 Framework application

Frameworks were applied with differing degrees of explicitness, which becomes particularly important with regard to underlying definitions of established frameworks. The ethical implications of the One Health approach for animals, for example, can vary depending on its interpretation. In a more general sense, it may remain human-centered, suggesting that animals should be considered in efforts to improve human health. For a stronger interpretation, however, the aim might be “improving human, animal, and environmental health” [(73), p. 6]. This interpretation implies that it is not sufficient that the animals are merely not stressed or harmed, but that they must experience a robust benefit (73). Several of the reviewed studies adopting the One Health concept assume the stronger interpretation, namely that well-structured AAS can simultaneously promote both human and animal health and flourishing, see for example Hediger et al. (16). Mignot et al. (35) further emphasize this perspective by drawing on the One Welfare concept derived from One Health and naming the robust benefits for the dog as a minimum requirement for ethical justification of AAS.

This review indicated that multiple ethical frameworks may coexist within individual studies, at times leading to conceptual conflation. Such conflation can affect both the structure of the argument and the interpretation of welfare findings. While negative and positive welfare are not mutually exclusive, a focus on negative welfare alone does not capture animal wellbeing, flourishing, or other articulations of positive animal welfare, including perspectives that encompass the full lifespan of the animals involved, addressing aging and death (108), multispecies work lives (15), and the animals' social relations (109).

One possible explanation for this pattern lies in methodological practice. Measures of negative welfare are generally more established and easier to operationalize, making them more readily measurable in empirical research (110). At the same time, however, the field has increasingly shifted toward positive animal welfare frameworks and perspectives that emphasize human-animal mutuality. A source of ambiguity concerns how human and animal benefits are balanced in the ethical justification of AAS. For example, Ayala et al. [(50), p. 12] argue that the absence of stress for the animal, “together with the wellbeing that the equine assisted therapy produces in the patients justifies its use.” This illustrates how the reported absence of indicators of negative animal welfare may be combined with reference to human benefit to justify AAS.

Overall, these observations highlight the need for greater transparency and reflexivity regarding the ethical framing of welfare assessments, as well as further methodological development of tools capable of assessing positive welfare in AAS contexts.

### 4.3 An overarching theme: animal roles and relational ethics

One overarching, ethically significant theme identified across the literature concerns the quality of human-horse and human-dog relationships and, more broadly, a relational focus in framing AAS practices. Ethical issues concerning human-animal relations are prominently articulated through concepts such as the handler-animal-client triad, which conceptualizes the relationships between clients, handlers, and animals.

Through the triadic model, the literature highlights different ethical and practical concerns. A recurring theme is the handlers' responsibility to recognize and respond to animals' behavioral signs of stress and wellbeing and to advocate for the individual animals involved in AAS, including ensuring that it is possible for them to withdraw from interactions and to gain new competences (7, 38, 71, 111, 112).

Accordingly, the literature frames adequate education and training for practitioners as crucial for safeguarding animal welfare, given the central role that handlers play in recognizing and responding to animals' needs (112). Within this framing, education may extend beyond recognizing welfare indicators to include advocacy for the animal and attention to concepts such as animal consent (5, 113). More broadly, AAS education may integrate not only findings from animal welfare science but also ethical reflection on genuine mutuality and animal consent, for example within One-Health-related approaches (73). Building on this perspective, we suggest that reflection on detachment—allowing animals to withdraw and maintaining distance—is also needed in AAS training and education, not as failure but as a potentially integral part of AAS. At the same time, attention to the quality of relations also emphasizes that responsibility for animal welfare cannot rest solely with individual practitioners but should involve collective professional standards and institutional conditions (83).

Beyond the quality of individual relationships, the literature suggests a shift in how animals' roles are framed in AAS. As Grandgeorge et al. (4) summarize, animals in animal-assisted work were often described in the past as instrumental to therapeutic success, whereas more recent literature frames them in relational terms. While this development may represent a revaluation of the animals' status, it raises new ethical questions about expectations of animals described as partners or team members rather than tools. At this point, it is important to distinguish between relatively broad or conceptually underspecified relational framings and more developed approaches in relational ethics. References to partnership, mutuality, or the human-animal bond do not in themselves clarify what makes a human-animal relationship ethically valuable or justified. More formal ethical approaches can make these normative assumptions more explicit by offering different perspectives on how relationships should be evaluated, for example by distinguishing between cooperation and exploitation (107) or between different forms of care-ethical relations, including asymmetrical relations and ideas of *philia*, friendship with animals (114). Reflections based on relational ethics and related approaches may therefore help to analyze not only the roles animals are expected to play in AAS practices, but also the ethical quality of the relations in which these roles are embedded (115, 116).

Importantly, relational framings require scrutiny. The broader relational turn in theories of human-animal relations has been described as a “mixed blessing” [(117), p. 316]: while relational approaches can decenter human interests and emphasize interdependence, they may also obscure asymmetries of power and privilege particular relationships or interests over others. Similar concerns are articulated within the AAS literature itself. For example, Glenk and Foltin [(8), p. 2] emphasize that handlers “must recognize the power differential within the triadic relationship and aim to protect their dog's optimal welfare despite the recipient's or handler's preferences.”

Scrutiny is also needed regarding how such relationships are interpreted within AAS research. Researchers have cautioned that AAS research may be subject to positive bias in evaluating the human-animal bond, for example due to expectations of beneficial outcomes (44), while processes of normalization may render ethical tensions less visible (5, 84). These concerns indicate that a relational focus alone does not resolve questions of power, welfare, or ethical justification. Although relational perspectives highlight important dimensions of welfare and ethics, empirical methods for assessing relationship quality (both animal-client and animal-handler) from an animal-centered perspective remain limited and should be developed further (118). Finally, the review suggests that the current welfare literature could benefit from greater engagement with the broader scope of contemporary animal ethics and politics, particularly regarding the extent to which animals are considered to be agents in work relations and political communities (15, 119).

## 5 Conclusion

This critical review mapped the variety of ethical approaches and assumptions present in the research on horse and dog welfare in animal-assisted services (AAS). By demonstrating how different framing may influence the interpretation and conclusions of welfare findings, we highlight the need for greater explicitness and transparency in presenting assumptions about animals' roles, as well as perspectives on animal use and potential mutual benefit.

The paper suggests, firstly, that a clearer articulation of these ethical framings—including both negative and positive animal welfare perspectives—will help position welfare studies along key ethical axes. Secondly, we recommend particular attention to how consistently ethical framings are used throughout a paper, and critical scrutiny of inconsistent applications that may for example create the impression that the absence of negative welfare findings is sufficient to confirm a positive welfare orientation. This, in turn, will enhance comparability and support the coherence of the literature, providing a more robust foundation for developing guidelines and policies aimed at improving—and potentially rethinking—animal-assisted services from animal-centered perspectives.

Thirdly, we recommend that authors of animal welfare studies in AAS should engage more explicitly in reflexivity regarding the normative assumptions they may bring to their research and include a reflexivity statement in their papers. Such statements, while more common in the social sciences, have also been recommended more broadly across the sciences (120, 121) and in the context of farmed animal welfare research specifically [(122), p. 7]. We suggest that their inclusion could further enhance transparency in this field.

The results of this critical review may also inform the further development and validation of welfare assessments, as well as evaluation of the coherence of animal welfare and animal ethics education in AAS by clearly distinguishing between perspectives that emphasize mutual human-animal benefit and those focused on avoidance of animal harm and stress.

## Footnotes

1 We thank one of the reviewers for suggesting this framing in terms of conceptual mismatch.
